# Methylseleninic acid elevates REDD1 and inhibits prostate cancer cell growth despite AKT activation and mTOR dysregulation in hypoxia

**DOI:** 10.1002/cam4.198

**Published:** 2014-02-07

**Authors:** Indu Sinha, Joshua E Allen, John T Pinto, Raghu Sinha

**Affiliations:** 1 Department of Biochemistry and Molecular Biology, Penn State College of Medicine, Penn State Hershey Cancer InstituteHershey, Pennsylvania; 2 Department of Medicine, Penn State College of Medicine, Penn State Hershey Cancer InstituteHershey, Pennsylvania; 3 Department of Biochemistry and Molecular Biology, New York Medical CollegeValhalla, New York

**Keywords:** Methylseleninic acid, prostate cancer, REDD1

## Abstract

Methylseleninic acid (MSeA) is a monomethylated selenium metabolite theoretically derived from subsequent *β*-lyase or transamination reactions of dietary Se-methylselenocysteine that has potent antitumor activity by inhibiting cell proliferation of several cancers. Our previous studies showed that MSeA promotes apoptosis in invasive prostate cancer cells in part by downregulating hypoxia-inducible factor HIF-1*α*. We have now extended these studies to evaluate the impact of MSeA on REDD1 (an mTOR inhibitor) in inducing cell death of invasive prostate cancer cells in hypoxia. In both PTEN+ and PTEN− prostate cancer cells we show that MSeA elevates REDD1 and phosphorylation of AKT along with p70S6K in hypoxia. Furthermore, REDD1 induction by MSeA is independent of AKT and the mTOR inhibition in prostate cancer cells causes partial resistance to MSeA-induced growth reduction in hypoxia. Our data suggest that MSeA induces REDD1 and inhibits prostate cancer cell growth in hypoxia despite activation of AKT and dysregulation of mTOR.

MSeA elevates REDD1 and AKT to promote cell death in invasive prostate cancer cells in hypoxia.

## Introduction

Hypoxia in solid tumors, and in particular those of the prostate, offers resistance to chemotherapy and radiation treatment modalities and can be a negative factor for survival in patients with prostate cancer 1. Reduced oxygen tension within the prostate induces the transcription factor, hypoxia-inducible factor (HIF)-1*α* 2. Upregulation of HIF-1*α* activates oncogenes and inactivates tumor suppressor genes which in combination may contribute to survival of prostate cancer cells and increase their metastatic potential 3. HIF-1*α* also enhances expression of genes coding for growth factors and their receptors, components of the apoptotic pathway, as well as cell-cycle regulators 4–6. Thus, HIF-1*α* is a major regulator of the hypoxic adaptive response of prostatic tumors, which makes HIF-1*α* a viable target for both chemopreventive and chemotherapeutic strategies. To date, ongoing clinical trials targeting HIF-1*α* are based primarily on blocking angiogenesis that is a major consideration in treatment of castration-resistant prostate disease 7,8. However, as with most chemotherapeutic strategies, these approaches appear to manifest with a number of inherent side effects. Chemoprevention strategies that perhaps target early destabilization of HIF may provide a more effective approach. However, caution must be drawn with these approaches as well since HIF-1*α*, despite its importance in tumor survival, is critical for regulating normal cells in response to transient conditions of hypoxia.

Expression of the transcription factor, REDD1 (regulated in development and DNA damage response-1) results in a negative regulation of the phosphatidylinositol 3-kinase (PI3K)- AKT/mammalian target of rapamycin (mTOR) pathway. Accordingly, REDD1 is downstream of PI3K 9,10 and regulates mTORC1 activity 11. Moreover, the regulation of HIF-1*α* by REDD1 is distinct from its ability to inhibit mTORC1 activity 12. Genetic ablation of REDD1 potentiates proliferation- and anchorage-independent growth particularly under hypoxic conditions.

Studies on human cancer cells have revealed that expression of REDD1 is downregulated in human cancers 12. By contrast, overexpression of REDD1 can be both protective and detrimental to cells under oxidative stress 13. REDD1 can be induced by a variety of stress conditions, such as hypoxia, ionizing radiation, and food deprivation or energy stress 13,14. Moreover, REDD1 can be induced by insulin in adipocytes through activation of MEK/ERK pathway. The effect of insulin appears to be associated with diminished REDD1 protein degradation, an event that is more pronounced under hypoxic condition in the presence of HIF-1*α* 15. Most studies link REDD1 to tumor suppression due to its contributions to apoptotic cell death 13 and to targeting forkhead box O transcription factors 16.

The exact function of REDD1 in prostate cancer is not completely understood. Schwarzer et al. 9 demonstrated a requirement of REDD1 gene for prostate tumorigenesis and another study 17 reported elevation in REDD1 protein-induced apoptosis in prostate cancer cells. It appears that prostate cells harboring wild-type p53 can upregulate expression of REDD1 in response to DNA damage, whereas in cells with mutated or dysfunctional p53, REDD1 is not targeted. By contrast, exposure of prostate cells to alkylating agents and hypoxia, REDD1 expression is induced by HIF-1*α* and can negatively regulate mTOR activity in prostate cancer cells.

The ability of reactive oxygen species (ROS) to promote HIF-1*α* stabilization suggests that the use of antioxidants can suppress tumorigenesis through modulation of HIF-1*α* 18. In a similar fashion, REDD1 can regulate production of ROS in mitochondria of embryonic fibroblast cells 12. Thus, as HIF-1*α* and REDD1 are redox-responsive proteins, changes in the intracellular redox environment with exogenous antioxidants would be expected to enhance their impact on apoptosis and growth inhibition.

Several experimental and epidemiology studies, as well as clinical intervention trials have supported the hypothesis that selenium-enriched diets can reduce the risk of prostate cancer 19–27. Important features of organoselenium compounds have been identified that relate directly to their chemopreventive properties particularly in prostate cancer cells, such as antioxidants, inhibitors of growth and androgen reactivity, regulators of signal proteins and apoptotic inducers, and modulators of gene expression 28–30. These and other anticancer mechanisms ascribed to organoselenium compounds are markedly dependent on their chemical forms and metabolic transformations. Chemopreventive mechanisms involving methylselenol as a regulator of redox-sensitive signal proteins and the metabolic conversions of selenoamino acids into seleno-keto acids, which function as histone deacetylase inhibitors have been reviewed elsewhere 31–33.

A promising anticancer agent methylseleninic acid (MSeA) has been shown to be effective in inhibiting prostate cancer growth in vitro and in vivo models 24,34,35. Recently, we showed that MSeA blocks growth of rat and human prostate cancer cells 24, an effect that has been associated with downregulation of HIF-1*α* even under hypoxic conditions. Reports by others show that inorganic sodium selenite can reduce HIF-1*α* and expression of vascular endothelial growth factor in melanoma cells 36 and that HIF-1*α* degradation in clear cell renal cell carcinoma by Se-methylselenocysteine is dependent on prolyl hydroxylase (PHD2) 37.

In the current investigation we show for the first time that MSeA elevates REDD1 expression in invasive prostate cancer cells under hypoxic conditions and maintains lower levels of HIF-1*α*. Additionally, we observe activation of AKT and mTOR that may in part explain growth inhibition of invasive prostate cancer cells in hypoxic condition.

## Material and Methods

### Cell lines and treatments

LNCaP cells were maintained in RPMI-1640 (Invitrogen, Carlsbad, CA), DU145 cells were maintained in Minimum Essential Medium (ATCC, Manassas, VA), and PC3 cells were grown in F-12K medium (ATCC). These prostate cancer cell lines were obtained from American Type Culture Collection, Manassas, VA. PC-3M cells (obtained from Dr. Isiah Fidler at M.D. Anderson Cancer Center, Houston, TX) were maintained in MEM (Invitrogen). REDD1+/+ and REDD1−/− cells (obtained from Dr. Leif Ellisen at Massachusetts General Hospital, Boston, MA) were maintained in Dulbecco's Minimum Essential Medium (Invitrogen). All media were supplemented with 10% FBS, and 1% penicillin–streptomycin solution unless stated otherwise. All cell types were routinely passaged weekly and maintained in 5% CO_2_ at 37°C. Prior to treatments, cells were incubated with 0.1% FBS overnight. Next-day treatments involved repletion of control cells with fresh medium containing 10% FBS alone and with MSeA (2.5–10 *μ*mol/L) and other agents (1 *μ*mol/L Wortmannin, 10 nmol/L rapamycin, 100 *μ*mol/L ascorbate, 2.5–5 *μ*mol/L sodium selenite) to experimental groups for 2 h, 4 h, 6 h or 18 h (overnight). For normoxic conditions, cells were treated in incubators, accessible to ambient air (∼21% O_2_ levels). For experiments performed under hypoxic conditions, cells were placed in MIC-101 Modular Incubator Hypoxia Chamber (Billups-Rothenberg Inc., Del Mar, CA) and perfused with a premixed standard gas containing 1% O_2_, 5% CO_2_, and 94% nitrogen (GTS-Welco, Reading, PA). The airspace within the chamber was perfused twice for 4 min each at a flow rate of 20–25 L/min at 2 psi with the premixed gas and the sealed chamber was placed in a 37°C incubator.

### Cell growth inhibition (MTT Assay)

Human prostate cancer cells or REDD1−/− and REDD1+/+ mouse embryonic fibroblasts (MEFs) were plated at a density of 10,000 cells/well in 96-well plates. On the following day, prostate cancer cells were incubated with MSeA (2.5–10 *μ*mol/L) or 2.5 *μ*mol/L sodium selenite for overnight in normoxic or hypoxic conditions as described above. The untreated and dimethyl sulfoxide (DMSO)-treated prostate cells or MEFs served as controls. 3-(4,5-dimethythiazol-2-yl)-2,5-diphenyl tetrazolium bromide (MTT) (50 *μ*g/well) was added to cell cultures containing phenol red-free plain medium under controlled lighting and incubated for 4 h at 37°C in the dark. The medium containing unreacted MTT was aspirated and DMSO solution was added to dissolve the insoluble formazan crystals and absorbance was read at 570 nm 38.

### Immunoblotting

Control and treated cells were harvested by scraping and washed with cold phosphate buffered saline (PBS)-containing protease inhibitors. Nuclear and cytosolic proteins were extracted as described previously 24. Equal amounts of protein (50 *μ*g) were separated on 8%, 10% or 15% SDS-PAGE gels depending upon the protein being studied and transferred to polyvinylidene fluoride membranes. Primary antibodies against AKT, phospho-AKT (Ser473), phospho-GSK3*α*/*β* (Ser21/9), cleaved-PARP (Asp214), HIF-1*β*, p70S6K, phosphorylated p70S6K, RPS6, phosphorylated-RPS6, 4E-BP1 (Cell Signaling, Danvers, MA), HIF-1*α* (R&D Systems, Minneapolis, MN), REDD1 (Proteintech, Chicago, IL), Lamin B and *β*-actin (Santa Cruz Biotechnology, Santa Cruz, CA) were reacted at 1:1000 with blots. The horseradish peroxidase (HRP)-conjugated anti-mouse, anti-rabbit or anti-goat secondary antibodies (Cell Signaling) were used at a dilution of 1:3000. Band expressions were developed using Pierce ECL reagents (Thermo Scientific, Rockford, IL) and the band densities were quantitated using ImageJ analysis (National Institute of Health, Bethesda, MD). The fold change in band densities of HIF-1*α* were normalized to the band densities of the respective Lamin B levels of the nuclear extracts in all samples. Fold change in band densities of phosphorylated proteins were normalized to the band densities of their respective native protein and to *β*-actin levels unless indicated otherwise. All Western blotting experiments were repeated two to three times and representative data are shown for each.

### GSH, cysteine, and methionine determinations

PC-3M cells were treated with 5 and 10 *μ*mol/L MSeA in 1% O_2_ for 2 h. Following, a wash in ice-cold PBS, cells were pelleted at 5000*g* for 10 min and the supernatant fraction removed. The pelleted cells were resuspended in ice-cold (4°C) metaphosphoric acid (5%) and rapidly vortexed to lyse cells and precipitate proteins. After 15 min on ice, samples were centrifuged at 5000*g* for 10 min and the supernatant solution was injected into an high-performance liquid chromatography for determination of glutathione (GSH) and concentrations of the sulfur-containing amino acids, cysteine, and methionine as previously described 39. Average values of three observations were determined for control and MSeA-treated PC-3M cells.

### Efficacy of MSeA in xenograft tumors

Athymic nude mice (males, 8 weeks of age) were subcutaneously inoculated with 5 × 10^5^ PC-3M-Luc (expressing luciferase) cells in PBS. These cells were generated in-house. Briefly, phoenix-Ampho cells were transfected by Lipofectin 2000 with pKS-neo-Luc, which encodes for the firefly luciferase gene under a constitutively active promoter and maintained under standard cell culture conditions. The medium containing the replication-deficient retrovirus was added to PC-3M cells in a 12-well plate format growing in log phase. Cells were selected by G418 (Geneticin, Life Technologies, Grand Island, NY) (600 *μ*g/mL) for 1–4 weeks until significant luminescence was observed following incubation with d-luciferin.

Mice were assigned to receive normal saline (*N* = 4) or MSeA (*N* = 5) via intra-peritoneal (i.p.) injections with the following doses 2.5 ppm (three injections on day 1, 4, and 6), 4 ppm (two injections on day 8 and 11), and 5 ppm (three injections on day 13, 15, and 19). The doses were based on our maximum-tolerated dose experiments and as reported by others 40. The aim of this experiment was to examine the efficacy of MSeA in a dose-escalation manner but we wanted to limit the selenium treatment to nontoxic doses (2.5–5 ppm) for mice. The nude mice were sacrificed on day 20. The tumor measurements were made on day 1 (baseline), 5, 10, and 20 posttreatment and tumor volumes were determined using the formula: 0.523 × *a*^2^ × *b*, where *a* is the smallest diameter and *b* is the largest. Body weights of the control and treated mice were also measured during treatment.

Tumor burdens were assessed by Bioluminescence imaging (BLI) using IVIS Spectrum (PerkinElmer, Waltham, MA) at baseline (tumor size approximately = 200 mm^3^), and at 7 and 20 days after multiple treatments with MSeA by i.p. (as described above). Briefly, the sterile anesthesia/d-luciferin aqueous solution (180 *μ*L) containing a dose of 130 mg/kg ketamine, 10 mg/kg xylazine, and 150 mg/kg potassium d-luciferin based on a mouse body weight of 20 g was injected i.p. into each mouse. Five minutes later, mice were laid down on the warm platform and imaging was performed for 5 sec. The total flux was determined as photons/sec. The mice were sacrificed 20 days post-treatment.

### Tumor cell proliferation (immunohistochemistry for Ki67)

The percent of Ki67-positive cells counted in triplicates in high-power fields (400×) was used as a measure of proliferation. Briefly, formalin-fixed paraffin-embedded prostate sections were hydrated, subjected to antigen retrieval and incubated with anti-Ki67 antibody (M7240; Dako North America Inc., Carpinteria, CA). Slides were developed with using Dako Envision™ +/HRP polymer detection system (K4001; Dako North America Inc.) and visualized with 3,3'-diaminobenzidine chromagen followed by hematoxylin counterstain.

### Statistical analysis

An analysis of variance (ANOVA) was performed to compare tumor weights, tumor volumes, body weights, BLI (photons/sec) within the MSeA and saline vehicle-treatment groups. Student's *t*-test was used to compare the vehicle-treated cells with various concentrations of MSeA, or rapamycin with and without MSeA in the MTT assay, and immunohistochemistry for Ki67. A value of *P* < 0.05 was considered statistically significant for these parameters.

## Results

### MSeA elevates expression of REDD1 and phosphorylation of AKT and p70S6K in invasive prostate cancer cells in hypoxia

**Figure 1:**
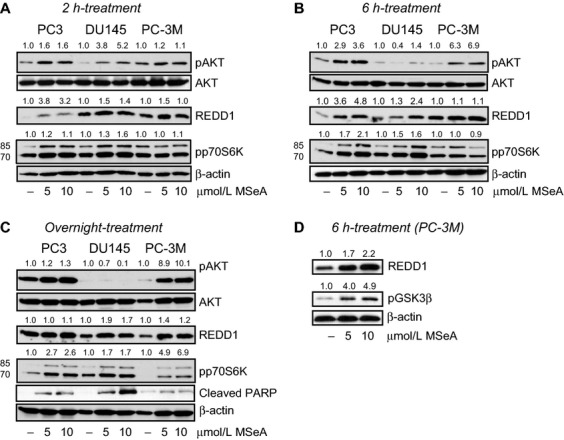
Protein expression changes in cytosolic fractions of PC3, DU145, and PC-3M cells following MSeA treatments for 2 h, 6 h and overnight in hypoxia. The three prostate cancer cell types were stimulated with 10% FBS following an overnight starvation (0.1% FBS) in the presence and absence of MSeA in hypoxia (1% O_2_). (A) Relatively increased levels of pAKT, REDD1 and pp70S6K (lower band) were observed after 2 h of MSeA treatment in invasive prostate cancer cells in hypoxia compared to respective untreated controls. (B) MSeA treatment after 6 h maintains elevated levels of pAKT in PC3 and PC-3M cells. REDD1 and pp70S6K levels induced by MSeA continue to increase in the three cell types at this time point. (C) An overnight exposure of invasive prostate cancer cells to MSeA maintained high levels of pAKT in PC3 and PC-3M cells while pAKT protein expression was marginally detectable in DU145 cells. The increase in REDD1 levels are more pronounced in the DU145 and PC-3M cells at this time point and pp70S6K expression was relatively higher in MSeA-treated cells as compared to untreated controls in each cell line. In addition, cleaved PARP appeared in a dose-dependent manner in each of the invasive prostate cancer cells following an overnight exposure with MSeA in hypoxia. (D) Following a 6 h treatment with MSeA in PC-3M cells, a concomitant dose-dependent elevation in pGSK3*β* expression was observed. Fold change in band densities of phosphorylated proteins were normalized to the band densities of their respective native protein and to *β*-actin levels. While for pp70S6K, pGSK3*β* and REDD1 the band densities were normalized to *β*-actin levels. FBS, fetal bovine serum; MSeA, methylseleninic acid; PARP, poly ADP-ribose polymerase; REDD1, regulated in development and DNA damage 1.

REDD1 regulates mTORC1 activity and the latter is critical for progression of prostate cancer disease. However, mTORC1 regulation by REDD1 has not been well studied in prostate cancer. We first investigated how MSeA impacts REDD1, AKT, and p70S6K (downstream target of mTORC1) in three different prostate cancer cell lines, DU145 (PTEN+), PC3, and PC-3M (PTEN−) at 2, 6 h and overnight (18 h) treatments with MSeA in hypoxia. Additionally, we used LNCaP cells but these did not survive in hypoxia and therefore were not included in further experiments. In DU-145 and PC-3M cells, MSeA upregulates REDD1 protein expression at all time points (Fig. 1A–C). However, in PC3 cells, REDD1 was increased by MSeA at 2 and 6 h time points but was stabilized by overnight treatment. In addition, pAKT levels are elevated following 2 h MSeA treatment in all the three cell types (Fig. 1A) and decrease only in DU145 cells at 6 h and overnight MSeA treatment (Fig. 1B and C). However, pAKT levels in control PC3 cells are increasing whereas in PC-3M control cells the pAKT levels are decreasing as a function of time in hypoxia. Similar observation has been reported by other investigators 41. Furthermore, MSeA-induced AKT activation in PC-3M cells was confirmed by phosphorylation of GSK3*β* in a dose-dependent manner after 6 h treatment (Fig. 1D). The phosphorylated p70S6K expression was elevated in all the prostate cancer cell lines at all time points following treatments with MSeA in hypoxia (Fig. 1A–C). The above events may play a role in MSeA-induced apoptosis (as measured by cleaved-PARP) in a dose-dependent manner in PC3, DU145, and PC-3M cells following overnight treatment (Fig. 1C).

### MSeA induces REDD1 expression independent of AKT in prostate cancer cells in hypoxia

**Figure 2:**
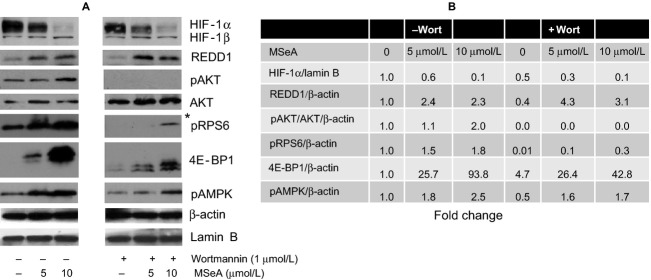
HIF-1*α*, REDD1, mTOR downstream proteins and pAMPK in PC-3M cells exposed to MSeA in presence of wortmannin in hypoxia. PC-3M cells were pretreated for 1 h with 1 *μ*mol/L wortmannin followed by 6 h treatment with MSeA in the absence or presence of wortmannin. (A) Nuclear extracts were processed for HIF-1*α* and Lamin B xpression levels and cytosolic fractions were analyzed for REDD1, native AKT, pAKT, pRPS6, 4E-BP1, pAMPK and *β*-actin protein levels. *Overexposed Western blot for visualization of pRPS6 in the presence of wortmannin. (B) Fold changes for target proteins estimated from immunoblots in (A) compared to control untreated PC-3M cells using ImageJ analysis. The fold change in band densities of HIF-1*α* were normalized to the band densities of the respective Lamin B levels of the nuclear extracts in all samples. The fold change in band densities of pAKT was normalized to the band densities of the respective native AKT and to *β*-actin levels. While for REDD1, pRPS6, 4E-BP1, and pAMPK the band densities were normalized to *β*-actin levels. HIF-1*α,* hypoxia-inducible factor-1 alpha; REDD1, regulated in development and DNA damage 1; MSeA, methylseleninic acid.

Previously published reports showed that REDD1 is downstream of PI3K-AKT 10,42 and is possibly required for prostate cancer progression 9. To determine if MSeA-induced changes in HIF-1*α*, REDD1, and mTORC1 are regulated by PI3K-AKT, we specifically decided to use PC-3M cells since these show elevated levels of pAKT and REDD1 following MSeA treatment at all time points. PC-3M cells were preincubated with wortmannin (1 *μ*mol/L) for 1 h prior to the 6 h treatment with MSeA in 1% oxygen environment. MSeA reduces HIF-1*α* levels in nuclear extracts in a dose-dependent manner both in absence and presence of wortmannin. However, decreases in HIF1*α* are more pronounced when AKT is blocked (Fig. 2). Moreover, MSeA markedly increases REDD1 expression both in the absence and presence of wortmannin. These results indicate that MSeA regulates REDD1 independent of AKT in hypoxia.

Concurrent with the above studies, the two downstream targets of mTORC1, RPS6, and 4E-BP1, were also investigated (Fig. 2), using wortmannin-induced blockade of PI3K in PC-3M cells in hypoxia. MSeA was able to increase phosphorylation of both RPS6 and 4E-BP1 in a dose-dependent manner in hypoxia. Despite AKT inactivation in PC-3M cells, MSeA was still able to initiate phosphorylation of RPS6 and hyper-phosphorylation 4E-BP1. However, the phosphorylation of RPS6 was much less pronounced in presence of wortmannin in these cells as observed when the blot was overexposed. Additionally, MSeA promoted phosphorylation of AMPK in a dose-dependent manner independent of PI3K-AKT block by wortmannin in hypoxia (Fig. 2). However, in presence of wortmannin the extent to which pAMPK was increased was less compared than that in the absence of wortmannin incubation in PC-3M cells treated with MSeA for 6 h in hypoxia. This may be explained in part by comparatively less anabolic stress occurring in these cells when AKT is inhibited.

### mTOR inhibition in prostate cancer cells causes partial resistance to MSeA-induced growth reduction in hypoxia

**Figure 3:**
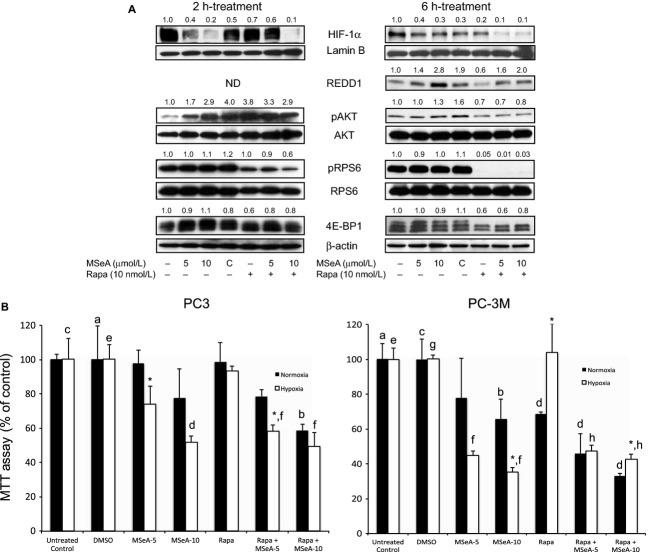
(A) HIF-1*α*, REDD1, pAKT, pRPS6, 4E-BP1 levels in MSeA-treated PC-3M cells in presence of rapamycin in hypoxia. PC-3M cells were pretreated for 1 h with rapamycin (10 nmol/L) before being exposed to 2 or 6 h treatment with MSeA. Nuclear extracts were processed for HIF-1*α* and Lamin B xpression levels and cytosolic fractions were analyzed for REDD1, native AKT, pAKT, pRPS6, 4E-BP1 and *β*-actin protein levels. MSeA reduced HIF-1*α* levels in a dose-dependent manner but in the presence of rapamycin moderate resistance occurs at the lower dose of MSeA (5 *μ*mol/L) at 2 h. At 6 h, HIF-1*α* levels were considerably reduced with the combined treatment. REDD1 levels were clearly upregulated by MSeA but downregulated by rapamycin treatment in 6 h under hypoxia. MSeA treatment of PC-3M cells in presence of rapamycin was able to rescue the REDD1 reduction. MSeA treatment clearly induces pAKT levels after 2 h and continues up to 6 h in hypoxia. However, in the presence of rapamycin, an increase was not evident at either time point compared to DMSO control. MSeA also increases levels of pRPS6 moderately (2 and 6 h) and rapamycin strongly blocks phosphorylation on RPS6 compared to DMSO control (C) at 2 h and was completely eliminated by 6 h. At 2 h, the hyper-phosphorylated bands on 4E-BP1 are upregulated following MSeA treatment but in the presence of rapamycin this band is less phosphorylated and completely disappears after 6 h combined treatment in PC-3M cells. ND: not determined. (B) Growth inhibition of prostate cancer cells treated with MSeA in the presence or absence of rapamycin when examined under normoxia or hypoxia. a versus b (*P* < 0.05), *N versus H: *P* < 0.05, c versus d, e versus f and g versus h: *P* < 0.01. The fold change in band densities of HIF-1*α* were normalized to the band densities of the respective Lamin B levels of the nuclear extracts in all samples. The fold change in band densities of pAKT and pRPS6 were normalized to the band densities of the respective native AKT and RPS6 and to *β*-actin levels. While for REDD1, and 4E-BP1 the band densities were normalized to *β*-actin levels. HIF-1*α,* hypoxia-inducible factor-1 alpha; REDD1, regulated in development and DNA damage 1; MSeA, methylseleninic acid; DMSO, dimethyl sulfoxide.

Since we observed a dose-dependent increase in pRPS6 levels in PC-3M cells, we next determined whether blocking mTOR function would alter survival factors and cell growth. PC-3M cells were treated with rapamycin (10 nmol/L) in presence and absence of MSeA. As shown in Figure 3A, MSeA inhibits HIF-1*α* at 2 and 6 h in the absence of rapamycin while in its presence no appreciable change in HIF-1*α* expression was observed in PC-3M cells compared to DMSO (vehicle control) at both incubation time points. Furthermore, in PC-3M cells that were treated simultaneously with MSeA and rapamycin (10 nmol/L) some resistance was observed in reducing HIF-1*α* levels with rapamycin at 5 *μ*mol/L MSeA. When cells were incubated for longer duration (6 h) under hypoxic conditions, HIF-1*α* levels were reduced considerably at both 5 *μ*mol/L and 10 *μ*mol/L doses of MSeA in the presence of rapamycin. As observed earlier, REDD1 levels in PC-3M cells were elevated by MSeA in (Fig. 1A and D), however, rapamycin treatment alone decreased REDD1 levels compared to those in DMSO control (Fig. 3B). This reduced effect of rapamycin was also reflected by MSeA + rapamycin combination when compared to MSeA treatment alone.

In the same experiment, MSeA-treated cells exhibited increased expression of pAKT, however, in cells treated with MSeA and rapamycin both pAKT and pRPS6 were reduced relative to that in DMSO controls. Under similar conditions, formation of hyperphosphorylated 4E-BP1 was reduced at 6 h by treatment with MSeA + rapamycin but not with MSeA alone, suggesting that rapamycin is effective in the PC-3M cells under hypoxic conditions.

In the next series of experiments, a survival assay was used to study the effects of MSeA alone and in combination with rapamycin in invasive prostate cancer cells. PC3 and PC-3M cells were treated with MSeA alone at 5 and 10 *μ*mol/L in normoxia or hypoxia in a 96-well MTT assay. Separate wells of these cells were also treated with DMSO and 10 nmol/L rapamycin alone and in combination (MSeA + rapamycin).

A dose-dependent growth inhibition was observed following treatments with MSeA in both cell lines more so in hypoxia than in normoxia yet for PC3 cells growth inhibition was significant at 5 *μ*mol/L but not at 10 *μ*mol/L between normoxia and hypoxia while the same trend was observed in PC-3M cells yet significant inhibition was observed only 10 *μ*mol/L MSeA (Fig. 3B). These data show that in hypoxia PC-3M cells are more sensitive to growth inhibition compared to PC3 cells. Growth inhibition was not observed with rapamycin alone in PC3 cells in normoxia or hypoxia. By contrast, a combination of rapamycin and MSeA (5 *μ*mol/L but not 10 *μ*mol/L) was more effective than rapamycin or MSeA alone in PC3 cells under both normoxic and hypoxic conditions. In PC-3M cells, growth inhibition was observed with rapamycin alone in normoxia but not in hypoxia. As previously noted, the combination of MSeA and rapamycin is more effective than MSeA alone in normoxia. However, the addition of rapamycin to MSeA did provide modest resistance to growth inhibition in MSeA-treated PC-3M cells in hypoxia, even though the cells do respond to rapamycin downregulation of mTORC1 as shown in Figure 3A.

### MSeA induces cysteine, methionine, and GSH levels in hypoxia

Within 2 h of hypoxic conditions alone, GSH levels in PC-3M cells increase (Table 1). When cells are treated with MSeA, GSH, cysteine, and methionine levels continue to rise in hypoxia. These data correlate with an increase in pp70S6K (Fig. 1A) and pRPS6 (Fig. 2) levels suggesting that the kinases involved in phosphorylation of p70S6K and pRPS6 may be influenced by the redox status of the cell. Therefore, we decided to examine this more closely in REDD1 MEFs.

**Table 1:** Cysteine, GSH, and methionine levels in PC-3M cells treated with MSeA in hypoxia.

Treatment	Cysteine (nmol/mg)	GSH (nmol/mg)	Methionine (nmol/mg)	Bound GSH (nmol/mg)	Free/bound ratio
Normoxia	4.19	44.83	0.71	0.88	52.76
Hypoxia	5.73	70.89	1.52	0.95	78.39
5* μ*mol/L MSeA	7.98	61.66	2.07	1.02	60.93
10 *μ*mol/L MSeA	12.68	72.23	2.46	0.84	86.11

Data are presented as average of three values. MSeA, methylseleninic acid.

### MSeA downregulates HIF-1α in REDD1 MEFs

**Figure 4:**
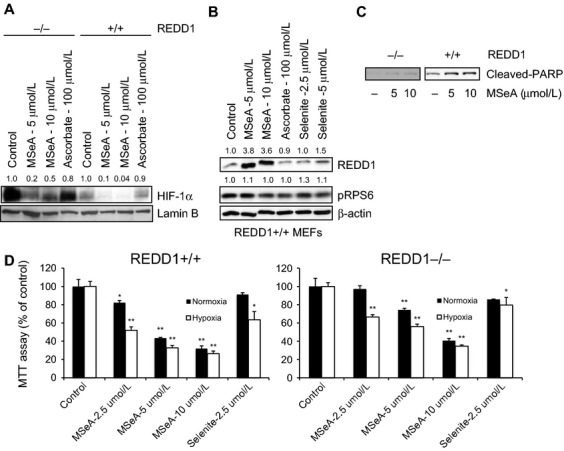
HIF-1*α* and REDD1 levels in REDD1 MEFs following treatments with MSeA, Selenite and ascorbate in hypoxia. Nuclear extracts were processed for HIF-1*α* and Lamin B expression levels and cytosolic fractions were analyzed for REDD1, pRPS6, cleaved-PARP and *β*-actin protein levels. (A) REDD1−/− cells express high levels of HIF-1*α* compared to REDD1+/+ cells and MSeA downregulates HIF-1*α* in both cell types. Ascorbate, on the other hand, does not show appreciable changes in HIF-1*α* in either cell type. (B) MSeA upregulates REDD1 protein expression in REDD1+/+ cells. Selenite also increases REDD1 levels but to less extent and ascorbate shows no change when compared to untreated controls. There are no appreciable changes in pRPS6 by either treatment. (C) REDD1+/+ cells are more sensitive compared to REDD1−/− cells for MSeA-induced apoptosis as shown by cleaved-PARP. (D) Growth inhibition of REDD1+/+ and REDD1−/− cells by MSeA in normoxia compared to hypoxia conditions. REDD1−/− cells are more resistant to growth inhibition by MSeA as compared to REDD1+/+ cells regardless of oxygen levels. *P < 0.05, **P < 0.001 as compared to respective normoxia and hypoxia controls. The fold change in band densities of HIF-1*α* were normalized to the band densities of the respective Lamin B levels of the nuclear extracts in all samples. The fold change in band densities of REDD1 and pRPS6 were normalized to *β*-actin levels. HIF-1*α,* hypoxia-inducible factor-1 alpha; REDD1, regulated in development and DNA damage 1; MSeA, methylseleninic acid; DMSO, dimethyl sulfoxide.

REDD1+/+ and REDD1−/− MEFs were treated with 5 and 10 *μ*mol/L MSeA or 100 *μ*mol/L ascorbate for 4 h in hypoxia. REDD1−/− cells express high levels of HIF-1*α* in hypoxia and MSeA treatment inhibits its expression much more efficiently than does treatment with ascorbate after 4 h (Fig. 4A). Reports by others in REDD1+/+ cells show that HIF-1*α* levels are elevated to a lesser extent in hypoxia compared to that in REDD1−/− cells 12. When REDD1+/+ cells are treated for 4 h with MSeA (5 and 10 *μ*mol/L), selenite (2.5 and 5 *μ*mol/L) and ascorbate (100 *μ*mol/L), MSeA induces REDD1 protein levels to greater extent than selenite and there was no change with ascorbate (Fig. 4B). There is no appreciable change in pRPS6 after all treatments in REDD1+/+ cells. Overnight treatment of REDD1+/+ and REDD1−/− cells with MSeA showed that PARP was cleaved although the extent of cleavage in REDD1−/− cells was visualized only upon overexposure of the blot (Fig. 4C). These results indicate that REDD1+/+ cells are more sensitive to apoptosis by MSeA in a dose-dependent manner compared to that of REDD1−/− cells. This observation is further supported by cell growth inhibition data shown in Figure 4D. Under both normoxic and hypoxic condition, MSeA in REDD1+/+ cells inhibited growth to a greater extent than in REDD1−/− cells. In comparison of cell survival between hypoxic and normoxic condition, both cell types demonstrated greater growth inhibition under hypoxic than in normoxic conditions. Although these data cannot be extrapolated to prostate cancer cells but it is evident from these experiments that MSeA is changing the redox state of cells in hypoxia and thereby increasing REDD1 and decreasing HIF-1*α*.

### MSeA reduces tumor growth of PC-3M-Luc cells in nude mice

**Figure 5:**
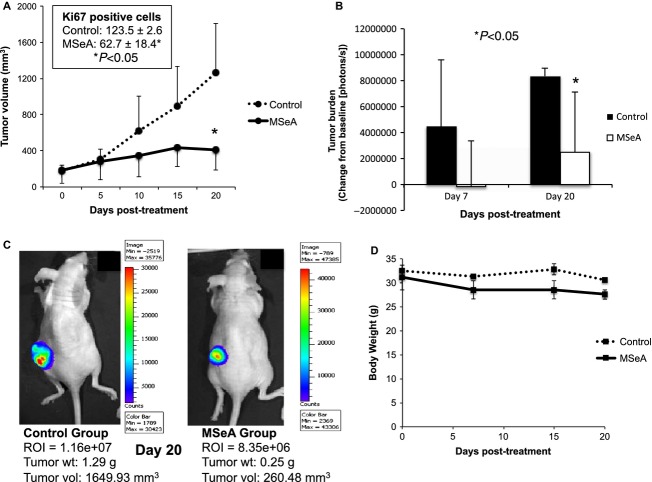
PC-3M-Luc tumor growth inhibition in MSeA-treated nude mice. PC-3M-Luc cells were injected s.c. into nude mice and treated with increasing doses of MSeA (i.p.) as described in materials and methods. (A) Tumor volume is reduced by MSeA treatment at the end of study (*P* < 0.05). Number of Ki67-positive cells was also reduced in tumor sections following MSeA treatment (inset). (B) Tumor burden as measured by change in luciferase activity from baseline is reduced following MSeA treatment (*P* < 0.05). (C) BLI measurements from representative saline and MSeA-treated PC-3M-Luc-bearing tumors and their respective characteristics. (D) Body weights of MSeA-treated nude mice are reduced compared to saline-treated control mice but the changes are not statistically significant. MSeA, methylseleninic acid; BLI, bioluminescence imaging.

Although, PC3 cells have been used for xenograft model for testing MSeA by oral route for about 49 days 43, our intent was to investigate the efficacy of MSeA against a highly invasive prostate cancer cell line (PC-3M). Therefore, we decided an i.p. route of treatment for a shorter duration. The PC-3M-Luc cells were injected subcutaneously into left flanks of nude mice. Tumor volumes showed a steady growth in control while the MSeA-treated tumors exhibited reduced volumes (*P* < 0.05) at the end of the experiment (Fig. 5A). Similarly, number of Ki67-positive cells was decreased in the tumors following MSeA treatment (control: 123.5 ± 2.6; MSeA: 62.7 ± 18.4, *P* < 0.05). Furthermore, tumor burdens in control and MSeA-treated mice were reflected by change (*P* < 0.05) in total luminescence flux by day 20 post-treatment (Fig. 5B). The BLI of representative mice from control and MSeA-treated groups are shown in Figure 5C. Body weights did not change significantly between control and treated groups during the short-term treatment with MSeA (Fig. 5D). At the end of the experiment, tumor weights were 0.91 ± 0.54 g in control and 0.44 ± 0.23 g in MSeA-treated mice. Despite the apparent changes in the mean values, differences in overall tumor weights were not statistically significant due to large variation between groups. These findings differ slightly from Li et al. 43 who showed that inhibition of tumor growth occurred after oral MSeA treatment for longer duration in xenograft models using LNCaP, DU145, and PC3 cells.

## Discussion

Numerous experimental and epidemiology studies, as well as clinical intervention trials have supported the hypothesis that selenium-enriched diets can reduce the risk of prostate cancer 19–27. Results from more recent Selenium and Vitamin E Cancer Prevention Trial (SELECT) failed to show a beneficial effect of selenomethionine on prostate cancer and vitamin E alone enhanced prostate cancer significantly 44. These data did raise the question of selenium's role in anticancer strategies but we have to realize that the form of selenium being tested is critical for a positive outcome and we need to investigate more potent selenium-containing agents as compared to selenomethionine. Previously, MSeA has successfully been tested as a promising anticancer agent in prostate cancer models 20,24. Our goal, for current investigation was to study the potential of MSeA in a highly metastatic prostate cancer model system and examine possible mechanism(s) by which it inhibits growth of these cells.

In the PC-3M xenograft study, using MSeA in a dose-escalation regimen our data demonstrate a decrease in tumor volume, tumor burden, and tumor weights. However, the overall tumor weights were not significantly reduced due to the short-term treatment and inter-animal variation. But PC-3M being a highly invasive prostate cancer model provides the opportunity to investigate the role of MSeA on HIF-1*α* regulation and its downstream targets.

In our previous report, we had documented MSeA-induced downregulation of HIF-1*α* in invasive prostate cancer cells 24. The mechanism of HIF-1*α* reduction was shown to involve a stabilization of the PHD domain. Recently, a similar finding demonstrated that Se-methylselenocysteine inhibited HIF-1*α* in renal cell carcinoma through a PHD2-dependent and Von-Hippel-Lindau-independent degradation mechanism 37. In the current investigation, our goal was to determine whether MSeA impacted REDD1 protein, a redox-sensitive downstream target of HIF-1*α*. REDD1 was initially identified as a hypoxia-regulated HIF-1*α* target gene involved in regulation of cell survival 13. Genetic studies revealed that REDD1 is a hypoxia-induced regulator of mTORC1 activity 11 and as a consequence in REDD1-/- cells, mTORC1 is not suppressed 45. Abrogation of REDD1 enhances tumorigenesis, a process that is associated with elevation of mitochondrial ROS which can stabilize HIF-1*α*. The regulation of ROS by REDD1 is mTOR-independent, which in the presence of antioxidants can restore normal levels of HIF-1*α*. Organoselenium compounds can affect the redox environment within cells as cited previously with GSH and we initially anticipated a decline in REDD1 levels following treatment with MSeA in hypoxia. Contrary to our original hypothesis, a 2-h exposure of cells to MSeA resulted in an upregulation of REDD1 protein expression in PTEN+ and PTEN− prostate cancer cell lines. Furthermore, MSeA was shown to sustain high levels of REDD1 in DU145 and PC-3M cells until the end of experiment in hypoxia. These results also correlate with induction of apoptosis in prostate cancer cells by MSeA in hypoxia as reflected by a dose-dependent cleavage of PARP.

In studies by Horak et al. 12, a loss of REDD1 expression was observed in breast cancer tissues as the disease progressed. However, a report by Schwarzer et al. 9 in PC3 prostate cancer cells showed that knockdown of REDD1 gene was able to sensitize these cells toward apoptosis. Thus, their studies suggested that overexpression of REDD1 would diminish apoptotic elimination of cells. By contrast, our data in DU145 and PC-3M clearly show that REDD1 protein expression is lower in the untreated-control prostate cancer cells but is elevated upon MSeA treatment in a dose-dependent manner. In fact, the elevated REDD1 levels in MSeA-treated prostate cancer cells may be a major contributor to cell death, a finding supported previously by Ben Sahra et al. 17 who showed that REDD1 is required for metformin-induced cell-cycle arrest and mTOR inhibition in prostate cancer cells.

Although, the experiment with REDD1 MEFs cannot be extrapolated to prostate cancer cells but using REDD1+/+ and REDD1−/− cells, modest resistance to cell death is observed in REDD1−/− cells as observed by cleaved-PARP and cell growth inhibition assays. This suggests that increased expression of REDD1 would facilitate MSeA-induced apoptosis in hypoxia.

Furthermore, MSeA-induced pAKT levels starting at 2 h post-treatment in hypoxia. These data corroborate with a concomitant increase in pGSK3*β* indicating AKT is functional in PC-3M cells. Studies by others 46 clearly demonstrate that GSK3*β* phosphorylates REDD1 and promotes its ubiquitination. Our studies indicate that elevated levels of pAKT results in inactivation of GSK3*β* through phosphorylation and, therefore, may explain why REDD1 accumulates in PC-3M cells following MSeA treatment in hypoxia. In addition, pp70S6K levels were elevated early on (at 2 h treatment) by MSeA indicative of mTORC1 activation in prostate cancer cells. Since PC3 and PC-3M cells are PTEN− it is expected that mTOR will not be completely inhibited in hypoxia 47.

Earlier studies 9,10 suggest that REDD1 is downstream of PI3K-AKT and that REDD1 inhibits mTOR function via TSC1/2 and 14-3-3 interactions 11. We investigated whether REDD1 is downstream of PI3K-AKT in invasive prostate cancer cells in hypoxic environment. Following treatment of prostate cancer cells with the PI3K inhibitor, wortmannin, MSeA was shown to induce a downregulation of HIF-1*α* and a concomitant upregulation of REDD1 protein in an apparent AKT independent manner. As a result, an increase in REDD1 expression by MSeA conceivably helped maintain low levels of HIF-1*α* expression in these cells. Mitochondrial REDD1 is most likely playing a role in regulation of HIF-1*α* as reported earlier 12, and this regulation by REDD1 is via a negative feedback loop, which is independent of its mTOR regulatory function.

Since AKT activation stimulates aerobic glycolysis in cancer cells and facilitates higher energy consumption 48, in the absence of HIF-1*α*, it is likely that elevated levels of pAKT and pp70S6K promote greater energy consumption under hypoxic conditions. This effect may cause tumor cells to undergo a hypermetabolic crisis. Following this crisis along with elevated pAMPK, the cells do not recover in spite of upregulation of survival signals in hypoxia and thus experience cell death. In other words, cells with activated AKT are more prone to ROS-induced stress resulting in apoptosis 49,50. It is noteworthy that the previous studies in human prostate cancer cells have shown that selenium can inhibit as well as activate AKT 51; however, our data unequivocally show that MSeA activates the AKT protein kinase (at early time point) concurrent with reduction in HIF-1*α* expression. This sustained oxidative stress may commit cells to apoptosis. Alternatively, MSeA-induced downregulation of HIF-1*α* in PC-3M cells may be allowing AKT activation via mTORC2 activation. These responses in metabolic pathways need to be examined in future experiments.

Active mTORC1 has a number of downstream biological effects including translation of mRNA via the phosphorylation of downstream targets (4E-BP1 and p70S6K). When PC-3M cells were treated with rapamycin (an inhibitor of mTORC1), we observed a time-dependent decrease in pRPS6 and hyper-phosphorylated band on 4E-BP1, which was not rescued by MSeA treatment. By contrast, rapamycin reduced REDD1 protein expression and MSeA treatment was able to reverse the inhibition. Our studies suggest that elevated levels of REDD1 by MSeA contribute to the growth inhibition in PC-3M prostate cancer cells under hypoxic conditions and that blocking mTORC1 would offer some resistance. In other words, elevation of both REDD1 and mTORC1 would facilitate enhanced cell death of PC-3M cells by MSeA in hypoxia. This is in contrast to PC3 cells in which the combination of rapamycin and MSeA offered greater growth inhibition compared to rapamycin or MSeA alone in both hypoxia and normoxia. In addition, a reduction in the levels of HIF-1*α* would be expected to promote a decrease in PC-3M cell growth.

In summary, our data demonstrate that MSeA induces REDD1 expression and promotes apoptosis in invasive prostate cancer cells in hypoxia. These findings are associated with a downregulation of HIF-1*α* and an activation of AKT along with mTORC1 signaling facilitating MSeA-induced growth inhibition. Understanding the mechanism(s) by which MSeA affects these pathways could be useful in inhibiting the transformation of prostate cells observed in prostatitis to those in benign prostatic hypertrophy (BPH) or prostatic intraepithelial neoplasia (PIN). The cellular transformations observed in these cells are associated with inflammation driven by HIF-1*α*-promoting growth events 52. MSeA may also be used in combination with chemotherapy agents for invasive and recurrent or resistant prostate cancers, which express high HIF-1*α*.
